# Effect of Niacin Supplementation During In Vitro Maturation on Fertilization Rate and Mitochondrial Competence of Vitrified and Nonvitrified Bovine Oocytes

**DOI:** 10.1155/vmi/9027270

**Published:** 2026-05-29

**Authors:** Mehdi Azari, Mojtaba Kafi, Davoud Eshghi, Ali Salehi, Fatemeh Farahmand

**Affiliations:** ^1^ Department of Clinical Sciences, School of Veterinary Medicine, Shiraz University, Shiraz, Iran, shirazu.ac.ir

**Keywords:** bovine, fertilization rate, niacin, oocyte maturation, vitrification

## Abstract

This study aimed to determine whether adding niacin to the oocyte maturation medium could enhance fertilization rate, mitochondrial distribution, and the expression of mitochondrial function–related genes in fresh and vitrified bovine oocytes. In Experiment 1, cumulus–oocyte complexes (COCs) were allocated into two groups for in vitro maturation: a control group and niacin‐supplemented (niacin group) oocytes. In Experiment 2, COCs were matured under the same conditions as in Experiment 1 and then vitrified, forming two additional groups: control vitrified (CV) and niacin‐supplemented vitrified (NV) groups. Fertilization rate, mitochondrial distribution, and gene expression were examined in the matured oocytes in both experiments. In Experiment 1, fertilization rate and gene expression were analyzed using a paired *t*‐test. In Experiment 2, fertilization rate and gene expression were analyzed by ANOVA with LSD post hoc test. Mitochondrial distribution patterns were assessed by Pearson’s chi‐square test in both experiments. In Experiment 1, niacin supplementation significantly improved fertilization rate and upregulated *TFAM*, *POLG*, and *NRF1* expression compared with the control group (*p* ≤ 0.05). In Experiment 2, although vitrification reduced fertilization rate overall, NV oocytes showed a tendency toward higher fertilization rate compared with the CV group (*p* = 0.08). Mitochondrial distribution in the CV group shifted toward a peripheral pattern, whereas niacin supplementation resulted in a more normal distribution. Vitrification decreased the expression of *COX1*, *POLG*, and *NRF1* (*p* ≤ 0.05) in the oocytes, but *NRF1* and *POLG* levels in the NV group were significantly higher than those in the CV group (*p* ≤ 0.05). In conclusion, niacin supplementation during in vitro maturation of bovine oocytes enhances fertilization competence by supporting mitochondrial distribution and mitochondrial gene expression, particularly under vitrification stress.

## 1. Introduction

In vitro embryo production (IVP) is now widely used to enhance the breeding of genetically selected livestock, particularly in cattle. Among the available techniques in IVP, cryopreservation of bovine oocytes is of particular importance because it enables embryo transfer at a convenient time for breeders, thereby promoting the efficient use of reproductive potential [1, 2]. Oocyte vitrification, a cryopreservation method characterized by a high concentration of cryoprotectants and a high cooling rate, is used to preserve genetically valuable animal resources. However, both in vitro and in vivo embryo development are impaired after oocyte vitrification. Optimizing in vitro oocyte maturation (IVM) culture conditions and vitrification procedures could enhance IVP efficiency [3]. One of the key factors influencing the effectiveness of IVP is the occurrence of oxidative stress during in vitro culture of oocytes [4]. Oxidative stress during in vitro culture, through the accumulation of reactive oxygen species (ROS), induces detrimental effects on cellular components, including DNA fragmentation, protein oxidation, lipid peroxidation, and mitochondrial damage [5, 6]. To minimize ROS‐induced damage, various antioxidants such as enzymes, amino acids, and vitamins have been incorporated into in vitro culture media to enhance oocyte maturation and embryo development [7, 8]. Nicotinic acid (niacin), a member of the vitamin B family, is a precursor of nicotinamide adenine dinucleotide [9]. Previous findings indicate that nicotinic acid possesses antioxidant properties that contribute to cellular membrane protection. It has also been shown to reduce lipid peroxidation biomarkers such as malondialdehyde (MDA) in rats [10, 11]. Moreover, nicotinic acid can stabilize free radicals and ROS. In addition, supplementation with nicotinic acid has been used in embryo culture to improve development to the blastocyst stage under heat shock conditions [12, 13]. Kafi et al. [14] reported that niacin improved IVP embryo quality and enhanced the tolerance of bovine oocytes to vitrification.

The quantity and pattern of mitochondrial distribution, along with ATP synthesis, are key factors influencing both the fertilization potential of the oocyte and the developmental competence of the embryo [15]. Several regulatory components also contribute to mitochondrial protection and biogenesis. Mitochondrial transcription factor A (*TFAM*) and nuclear respiratory factor 1 (*NRF1*) regulate mitochondrial DNA transcription and replication. Expression of the *POLG* gene is also involved in mitochondrial DNA transcription. Cytochrome c oxidase I (*COX1*), a respiratory chain protein encoded by mitochondrial DNA, may serve as an indirect marker of mitochondrial DNA activity and possibly its abundance [16]. To our knowledge, no study has examined the effect of adding niacin to the oocyte culture medium on fertilization rate and mitochondrial function in vitrified and nonvitrified bovine oocytes. Therefore, the present study was designed to evaluate the effect of niacin supplementation during IVM on (1) fertilization rate and (2) cryotolerance and mitochondrial distribution in vitrified and nonvitrified bovine oocytes. The expression patterns of genes associated with mitochondrial activity were further examined in vitrified and nonvitrified oocytes.

## 2. Materials and Methods

### 2.1. Ethical Considerations

This research received approval from the animal research committee of the School of the Veterinary Medicine, Shiraz University (98GCB1M370068).

### 2.2. Chemicals and Culture Media

The media and chemicals used in the present study were obtained from Sigma‐Aldrich (St. Louis, MO, USA), unless stated otherwise.

### 2.3. Experimental Design

**FIGURE 1:**
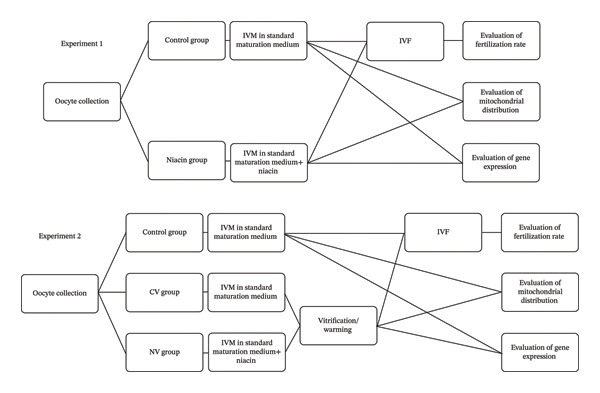
Experimental design.

The study was conducted in the IVF Laboratory at the School of Veterinary Medicine, Shiraz University, Iran. In Experiment 1, IVM was performed in a maturation medium containing 1 mM niacin. The optimal niacin concentration was selected based on the highest maturation rate after exposure to 0.5, 1, and 2 mM during IVM. Mitochondrial distribution and gene expression were assessed at the end of maturation, and the fertilization rate was subsequently evaluated. In Experiment 2, the effects of niacin on oocyte cryotolerance and the developmental competence of vitrified matured oocytes were examined. Figure 1 presents the study design for Experiments 1 and 2.

### 2.4. Oocyte Recovery

Bovine ovaries were collected at a local slaughterhouse immediately after slaughter and transported to the laboratory within 2‐3 h in physiological saline (NaCl) at 33°C–35°C. Cumulus–oocyte complexes (COCs) were retrieved from antral follicles (2–8 mm in diameter) using aspiration with a 20‐gauge needle attached to a 10‐mL syringe. After evaluation under a stereomicroscope, only COCs with at least three layers of compact cumulus cells and homogeneous cytoplasm (good and excellent quality) were selected for further procedures [17].

Experiment 1. Effect of niacin supplementation during in vitro maturation on mitochondrial distribution, gene expression, and fertilization rate.

### 2.5. In Vitro Maturation of the Oocytes

Selected COCs were washed three times in washing medium (TCM‐199 supplemented with 10% fetal calf serum [FCS]) and then matured in an IVM medium containing TCM‐199 (Sigma M4530) supplemented with 10% heat‐treated FCS, 0.1 IU/mL recombinant human FSH (Follitrope, LG Life Sciences, South Korea), 5 IU/mL highly purified hCG (Karma Pharmatech GmbH, Germany), and 50 µg/mL gentamicin. The medium was supplemented either with 1 mM niacin (niacin group) or with no niacin (control group). Groups of 40–50 COCs were transferred into four‐well dishes (NuncTM, Denmark) containing 500 µL of equilibrated maturation medium and incubated at 38.5°C with 5% CO2 and 90% humidity for 24 h [14]. The experiment was performed in five biological replicates.

### 2.6. Assessment of Mitochondrial Distribution in the Oocytes

Following each IVM replicate, 5–10 COCs in each group were mechanically denuded using a pipette under a stereomicroscope. To assess mitochondrial distribution patterns, oocytes were stained with rhodamine 123 (cat no. R8004, Sigma‐Aldrich, USA) as described previously [18]. After incubation in TCM‐199 containing 10 μg/mL rhodamine 123 for 20 min at 37°C with 5% CO2, the oocytes were washed, mounted on slides under cover slips, and examined immediately under UV light (405–435 nm) using a fluorescence microscope (Primo Star, Zeiss, Germany). Mitochondrial distribution was classified according to Liu et al. [19] as follows: (i) uniform distribution, with mitochondria dispersed throughout the cytoplasm; (ii) aggregated distribution, with mitochondria clustered irregularly; and (iii) peripheral distribution, with mitochondria localized near the plasma membrane. The experiment was performed in five independent replicates, and 54 control oocytes and 53 niacin‐treated oocytes were evaluated.

### 2.7. RNA Extraction

After IVM, RNA was extracted from 45 to 50 COCs in each group using the RNeasy Micro Kit (cat no. 74004, Qiagen, Hilden, Germany) according to the manufacturer’s instructions. RNA quantity and purity were assessed for each sample using a Nanodrop spectrometer.

### 2.8. Real‐Time Polymerase Chain Reaction

Following RNA extraction, reverse transcription (RT) was performed using the RevertAid H Minus First Strand cDNA Synthesis Kit (K1632, Fermentas, Germany). The expression levels of TFAM, POLG, COX1, and NRF1 were evaluated using real‐time PCR. GAPDH served as the internal reference gene. Primer sequences are listed in Table 1. Each PCR well contained 5 µL of Power SYBR Green PCR Master Mix, 1 µL of forward and reverse primers (each at 5 pM), 2 µL of cDNA (12.5 ng/µL), and 11 µL of water, resulting in a final volume of 20 µL. Reactions were performed on an Applied Biosystems StepOne real‐time PCR system with the following cycling protocol: initial denaturation at 95°C for 10 min; 40 cycles of 95°C for 15 s and 60°C for 1 min; and melting curve analysis at 95°C for 15 s, 60°C for 15 s, and 95°C for 15 s. Gene expression in the treatment group was compared with the control group using the 2^−ΔΔCt^ method. The experiment was performed in three biological replicates.

**TABLE 1:** Primers used for real‐time RT‐PCR of different genes.

mRNA	Sequence of primers	Product size	References
TFAM	F1 (5‐GTCGACTGCGCTATCCCTTT‐3) R1 (5‐TTTGCATCTGGGTTCTGAGC‐3)	148	[20]

POLG	F1 (5‐AGTCCCAGAGCAAAGCCAAC‐3) R1 (5‐TGTTGCCCTTGACAAACAGC‐3)	150	[20]

COX1	F1 (5‐AAATAATATAAGCTTCTGACTCC‐3) R1 (5‐TCCTAAAATTGAGGAAACTCC‐3)	148	[20]

NRF1	F1 (5‐CCCAAACTGAGCACATGGC‐3) R1 (5‐GTTAAGTATGTCTGAATCGTC‐3)	162	[20]

GAPDH	F1 (5‐GCCGTAACTTCTGTGCTGTG‐3) R1 (5‐AATGAAGGGGTCATTGATGG‐3)	150	[20]

### 2.9. In Vitro Fertilization

In vitro maturation of COCs was conducted as described earlier. Matured COCs in the control (*n* = 181) and niacin (*n* = 179) groups were transferred into 500 µL of fertilization medium (modified Tyrode’s medium supplemented with 0.27 mg/mL caffeine, 10 mg/mL heparin, 50 mg/mL hypotaurine, 6 mg/mL BSA, 1 mg/mL sodium pyruvate, 1.12 mg/mL sodium lactate, and 2.2 mg/mL sodium bicarbonate) in four‐well dishes (NuncTM, Denmark) at 30–40 COCs per well. Frozen semen previously validated for high fertility and acceptable IVP was thawed and processed using a swim‐up procedure. Spermatozoa were added at a concentration of 106/mL. Spermatozoa and COCs were coincubated at 38.5°C with 5% CO2 and maximum humidity for 22 h. The experiment was conducted in five biological replicates.

### 2.10. Evaluation of the Fertilization Rate

To assess fertilization, presumptive zygotes were mechanically denuded 18–20 h after insemination, mounted on glass slides under coverslips, and fixed with ethanol:acetic acid (3:1). After 24 h, slides were stained with 1% aceto‐orcein and examined using phase‐contrast microscopy. Zygotes were classified as fertilized (two pronuclei), unfertilized (no pronuclei or sperm and no second polar body), polyspermic (more than two pronuclei), or abnormal (other atypical nuclear configurations) [20].

Experiment 2. Effect of niacin supplementation to IVM medium on fertilization rate, mitochondrial distribution, and gene expression after vitrification.

### 2.11. Oocyte In Vitro Maturation and Vitrification

Following IVM, groups of 3–5 matured oocytes with fully expanded cumulus cells were placed in equilibration medium (7.5% dimethyl sulfoxide [DMSO] and 7.5% ethylene glycol [EG] in holding medium [HM; TCM‐199–HEPES + 20% FCS]) for 9 min at room temperature and then moved to vitrification medium (15% DMSO, 15% EG, and 0.5 mol/L sucrose in HM) at 25°C for 45 s. Oocytes were loaded onto Cryotop tips (Kitazato Supply Co., Tokyo, Japan) and plunged into liquid nitrogen.

After two weeks, vitrified oocytes were warmed by immersing the Cryotop tip into HM containing 1 M sucrose for 1 min at 37°C, followed by exposure to HM with 0.5 M sucrose for 3 min, and finally transferred to HM for 5 min [21].

### 2.12. In Vitro Fertilization

After IVM, oocytes were randomly allocated into two groups.

• Control vitrified (CV): oocytes matured in standard IVM medium and then vitrified (*n* = 151).
• Niacin vitrified (NV): oocytes matured in IVM medium containing 1 mM niacin and then vitrified (*n* = 197).

Twenty‐four hours before thawing vitrified oocytes, nonvitrified control oocytes (*n* = 169) were matured in vitro. Oocytes from all groups were then transferred into 500 µL of fertilization medium (modified Tyrode’s medium) in four‐well dishes, and high‐quality spermatozoa were added and coincubated at 38.5°C with 5% CO2 and 90% humidity for 22 h. Fertilization rate was assessed using the aceto‐orcein staining method described in Experiment 1.

After warming, mitochondrial distribution and expression of *TFAM*, *POLG*, *COX1*, and *NRF1* were evaluated using real‐time PCR as described in Experiment 1 [21]. Mitochondrial distribution was assessed in 54, 50, and 44 oocytes in the control, CV, and NV groups, respectively. The study design of Experiment 2 is presented in Figure 1.

Statistical analysis was performed using a computer‐aided software package (IBM SPSS Statistics, Version 22 for Windows). The Kolmogorov–Smirnov test was used to assess normality. In Experiment 1, fertilization rate and gene expression were analyzed using paired *t*‐tests. In Experiment 2, one‐way analysis of variance (ANOVA) followed by the LSD test was used to compare fertilization rate and gene expression. Mitochondrial distribution in Experiments 1 and 2 was analyzed using Pearson’s chi‐square test. Fisher’s exact test was applied when expected frequencies were low. Data are presented as mean ± SD. A *p* value of 0.05 or less was considered statistically significant.

## 3. Results

Niacin supplementation to the IVM medium improved nuclear maturation of oocytes (Table 2). The highest maturation rate was observed in the 1 mM group (87.2 ± 5.3%), which was significantly higher than the rates in the other groups. Based on these findings, 1 mM was selected as the optimal concentration for the present study.

**Table 2:** Mean (±SD) percentages of maturation following addition of niacin at different concentrations to the maturation media.

Groups	*N*	Mature, *n* (%)	Immature, *n* (%)	Abnormal, *n* (%)
Control	142	109 (76.1 ± 4.8)^a^	22 (15.2 ± 6.5)^a^	11 (8.5 ± 7.4)
0.5 mM	140	109 (78.9 ± 5.8)^a^	21 (15.3 ± 4.7)^a^	10 (5.7 ± 5.8)
1 mM	133	117 (87.2 ± 5.3)^b^	6 (4.5 ± 1.6)^b^	10 (8.1 ± 5.5)
2 mM	146	95 (65.1 ± 2.07)^c^	36 (24.3 ± 4.7)^c^	15 (10.4 ± 4.4)

*Note:* Values in the same column with different superscripts are significantly different (*p* ≤ 0.05).

Experiment 1. Effect of niacin supplementation to IVM medium on fertilization rate, mitochondrial distribution, and gene expression.

**FIGURE 2:**
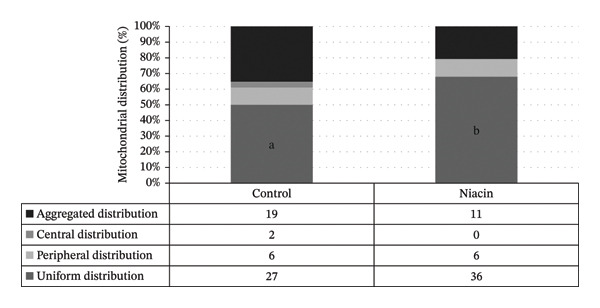
The pattern of mitochondrial distribution in oocyte after IVM (Experiment 1). The numbers in the table represent the number of oocytes. Different superscript letters in the uniform distribution pattern showed a tendency toward significant difference between groups (*p* = 0.06).

The results of Experiment 1 are summarized in Table 3. The mean (±SD) fertilization rate was higher in the niacin‐supplemented group (76.4 ± 6.8%) than in the control group (60.1 ± 5.3%) (*p* ≤ 0.05). Representative mitochondrial distribution patterns are shown in Figure 2. The number of oocytes displaying a uniform mitochondrial distribution pattern was higher in the niacin‐treated group (68%) than in the control group (50%) (*p* = 0.06).

**TABLE 3:** Number and mean percentage (±SD) of fertilized oocytes following the supplementation of niacin to the maturation medium (Experiment 1).

Groups	*n*	Fertilized	Unfertilized	Polyspermy	Abnormal
Control	181	110 (60.1 ± 5.3)^a^	60 (33.1 ± 7.3)^a^	8 (4.4 ± 3.8)	3 (1.6 ± 2.2)
Niacin	179	135 (76.4 ± 6.8)^b^	39 (20.7 ± 8.4)^b^	2 (0.9 ± 1.2)	3 (1.9 ± 3.1)

*Note:* Values in the same column with different superscripts are significantly different (*p* ≤ 0.05).

**FIGURE 3:**
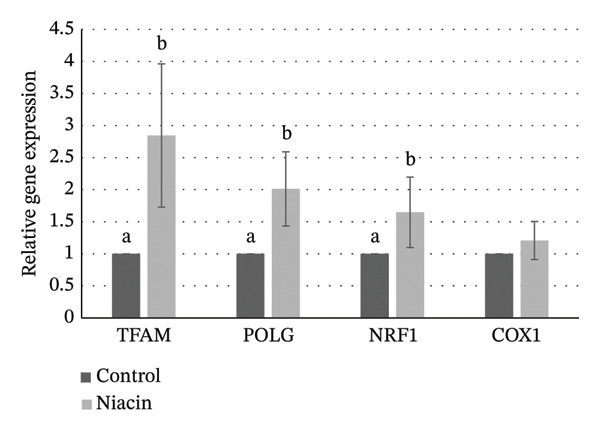
Relative expression levels of selected genes (*TFAM*, *POLG*, *NRF1*, and *COX1*) in MII oocytes (Experiment 1). Different superscript letters for the same gene indicate significant difference (*p* ≤ 0.05).

Expression levels of *TFAM*, *POLG*, *NRF1*, and *COX1* were evaluated by real‐time quantitative PCR. *TFAM*, *POLG*, and *NRF1* expression levels were significantly higher in niacin‐supplemented oocytes compared with control oocytes (*p* ≤ 0.05; Figure 3). No significant difference was observed in *COX1* expression among groups (*p* > 0.05).

Experiment 2. Effect of niacin supplementation to IVM medium on fertilization rate, mitochondrial distribution, and gene expression after vitrification.

As presented in Table 4, the fertilization rate was significantly higher (*p* ≤ 0.05) in the control group (70.8 ± 2.8%) compared with the CV group (27.2 ± 12.4%) and the NV group (36.05 ± 11.2%). Niacin supplementation tended to increase the percentage of normally fertilized oocytes in the NV group compared with the CV group (*p* = 0.08). The percentage of degenerated oocytes was significantly higher in the CV group compared with the other groups (*p* ≤ 0.05).

**TABLE 4:** Mean percentage (±SD) and number of fertilized oocytes following the addition of niacin to the maturation medium after vitrification (Experiment 2).

Group	*N*	Fertilized	Unfertilized	Polyspermy	Degenerated
Control	169	120 (70.8 ± 2.8)^a^	40 (24 ± 8.1)^a^	6 (3.5 ± 5.3)	3 (1.6 ± 2.4)^b^
CV	151	39 (27.2 ± 12.4)^b^	91 (59.4 ± 6.9)^b^	6 (3.6 ± 3.6)	15 (9.6 ± 4.2)^a^
NV	197	68 (36.05 ± 11.2)^b^	109 (53.6 ± 12.2)^b^	14 (6.9 ± 5.5)	6 (3.3 ± 3.2)^b^

*Note:* CV: control vitrified group; NV: niacin vitrified group. Values in the same column with different superscripts are significantly different (*p* ≤ 0.05).

**FIGURE 4:**
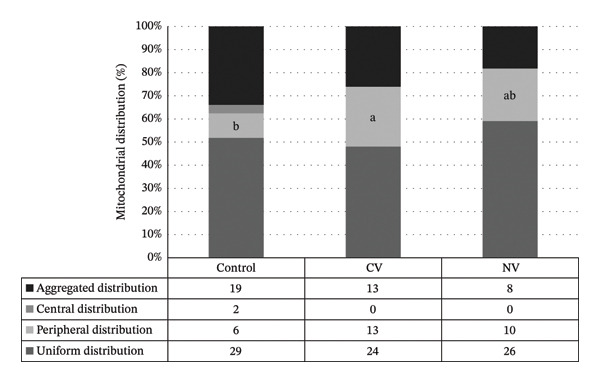
The pattern of mitochondrial distribution in oocyte after vitrification (Experiment 2). The numbers in the table indicate the number of oocytes. CV: control vitrified group; NV: niacin vitrified group. Different superscript letters in the same pattern indicate significant difference (*p* ≤ 0.05).

Mitochondrial distribution patterns in Experiment 2 are shown in Figure 4. No significant differences were detected in the percentage of uniform mitochondrial distribution among groups (*p* > 0.05). Oocytes in the CV group exhibited a significantly higher peripheral distribution compared with the control group (26.0% vs. 11.12%; *p* ≤ 0.05).

**FIGURE 5:**
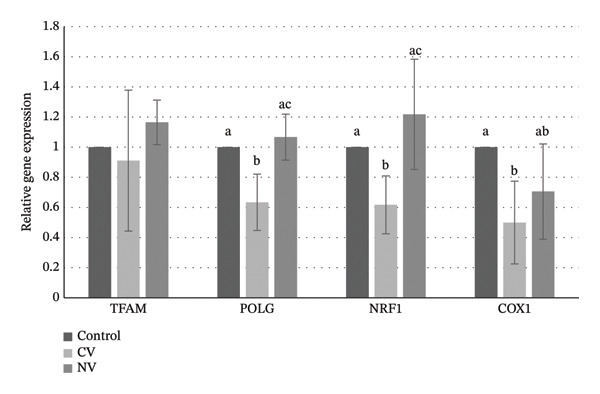
Relative expressions levels of selected genes (*TFAM*, *POLG*, *NRF1*, and *COX1*) in MII oocytes (Experiment 2). Different superscript letters for the same gene indicate significant difference (*p* ≤ 0.05). CV: control vitrified group; NV: niacin vitrified group.

Expression levels of *POLG*, *NRF1*, and *COX1* were downregulated in oocytes from the CV group compared with the control group (*p* ≤ 0.05), while no significant differences were observed between the NV and control groups. *NRF1* and *POLG* levels in the NV group were significantly higher than those in the CV group (*p* ≤ 0.05; Figure 5). *TFAM* expression did not differ significantly among groups.

## 4. Discussion

In the present research, we showed that incorporating niacin into the maturation medium may enhance the fertilization rate of bovine oocytes. We also observed that supplementation with 1 µM niacin moderately improved the cryotolerance of bovine oocytes. Although mitochondrial distribution did not differ between the CV and NV groups, the upregulation of *POLG* and *NRF1* in NV oocytes may partially account for the moderate improvement in fertilization. These findings appear to reflect enhanced mitochondrial function through improved mitochondrial dynamics and increased expression of genes involved in mitochondrial protection, as well as the regulation of mitochondrial DNA transcription and replication. However, these interpretations require further validation before causal relationships can be confirmed.

We previously reported [14] that MDA levels were significantly reduced when niacin was added to bovine oocyte culture medium after 24 h. MDA represents a stable end product of lipid peroxidation; thus, reductions in MDA may indicate decreased ROS. ROS may interact with intracellular macromolecules and induce lipid peroxidation and cellular damage [6, 22]. Several investigations have shown that niacin not only decreases lipid peroxidation but also elevates glutathione levels, likely due to its antioxidant capacity [12, 23, 24]. Recent work has also suggested that specific gene expression levels may serve as indicators of oocyte quality [25, 26]. Findings from Experiment 1 demonstrated that transcript levels of *TFAM*, *NRF1*, and *POLG* were significantly higher in niacin‐supplemented oocytes than in control oocytes. These observations suggest that niacin may enhance the quality of in vitro matured oocytes; nonetheless, the mechanisms underlying these changes need further investigation.

Oocyte cryopreservation remains an important tool for conserving genetic resources in farm animals and endangered species [27–30]. Cryopreservation may induce cytoskeletal injury by disrupting actin and microtubule networks [31], and it can impair mitochondrial function in bovine and human oocytes [32–34]. Numerous studies have shown that cryopreservation methods influence mitochondrial distribution, a factor essential for proper oocyte and embryo development. Migration of mitochondria toward the central region of the oocyte and the acquisition of a uniform mitochondrial distribution are considered markers of cytoplasmic maturation [19]. In Experiment 1, the percentage of oocytes with uniform mitochondrial distribution was higher in the niacin‐supplemented group than in controls, which may help explain the higher fertilization rates observed in this group. Nevertheless, while this association is suggestive, the present data do not provide direct evidence that mitochondrial distribution fully accounts for the observed fertilization differences.

In Experiment 2, peripheral mitochondrial distribution was significantly higher in oocytes from the CV group than those from the nonvitrified group, likely reflecting cytoskeletal disruption during vitrification and impaired mitochondrial migration. These observations align with previous reports showing reduced fertilization rates in vitrified oocytes relative to nonvitrified controls. Reduced cleavage and impaired embryo development after oocyte cryopreservation have been linked to zona hardening and cytoplasmic injury [14, 35, 36]. In the earlier work, we also recorded higher nuclear maturation and embryo production rates in niacin‐treated bovine oocytes compared with untreated oocytes, which was attributed to improved tolerance to vitrification through reduced lipid peroxidation [14]. Findings from Experiment 2 of the present study indicate that although niacin supplementation improved several indicators of cryotolerance, fertilization rates did not differ significantly between niacin‐treated vitrified and nonvitrified groups and showed only a moderate positive effect. This pattern suggests that factors beyond mitochondrial distribution and gene expression may contribute to variability in fertilization outcomes.

The transcript levels of *COX1* and *NRF1* are considered critical for oocyte developmental competence, and reduced expression of these genes has been reported in bovine oocytes that fail to cleave [37]. In addition, *TFAM*, *NRF1*, and *POLG* play essential roles in regulating mitochondrial DNA transcription and replication [16, 25, 38]. In our study, expression levels of *TFAM*, *NRF1*, and *POLG* were lower in vitrified control oocytes, while no significant difference was found between vitrified oocytes treated with niacin and nonvitrified controls. However, the level of expression of *POLG* and *NRF1* genes in the NV oocytes showed a significant increase as compared to the CV oocytes. This can partially explain the tendency in higher fertilization rate in NV oocytes than the CV oocytes. *NRF1* and *POLG* regulate mitochondrial DNA transcription and replication [16]. These findings support the beneficial influence of niacin on mitochondrial activity during vitrification. Consistent with these observations, Amoushahi et al. [38] reported lower cytochrome c oxidase activity, decreased mitochondrial DNA copy number, and reduced *TFAM* expression in vitrified mouse oocytes compared with nonvitrified controls.

## 5. Conclusions

In conclusion, this study suggests that niacin supplementation in the oocyte maturation medium may enhance mitochondrial distribution and increase the expression of mitochondrial genes involved in oocyte maturation. While the findings indicate potential improvements in oocyte quality and cryotolerance, the underlying mechanisms remain incompletely understood. Future research should aim to clarify the molecular pathways through which niacin influences mitochondrial function and to determine its potential role in improving fertilization and developmental competence in cryopreserved oocytes.

## Funding

This study was supported by Shiraz University, 98GCB1M370068.

## Conflicts of Interest

The authors declare no conflicts of interest.

## Data Availability

The data that support the findings of this study are available from the corresponding authors upon reasonable request.
